# Microneedles: A Versatile Drug Delivery Carrier for Phytobioactive Compounds as a Therapeutic Modulator for Targeting Mitochondrial Dysfunction in the Management of Neurodegenerative Diseases

**DOI:** 10.2174/1570159X20666221012142247

**Published:** 2024-02-03

**Authors:** Akshay Bandiwadekar, Kartik Bhairu Khot, Gopika Gopan, Jobin Jose

**Affiliations:** 1 Department of Pharmaceutics, NGSM Institute of Pharmaceutical Sciences, NITTE (Deemed-to-be University), Mangalore, 575018, India

**Keywords:** Neurodegenerative diseases, mitochondrial dysfunction, blood-brain barrier, phytobioactive compounds, microneedles, bioavailability

## Abstract

Neurodegenerative disease (ND) is the fourth leading cause of death worldwide, with limited symptomatic therapies. Mitochondrial dysfunction is a major risk factor in the progression of ND, and it-increases the generation of reactive oxygen species (ROS). Overexposure to these ROS induces apoptotic changes leading to neuronal cell death. Many studies have shown the prominent effect of phytobioactive compounds in managing mitochondrial dysfunctions associated with ND, mainly due to their antioxidant properties. The drug delivery to the brain is limited due to the presence of the blood-brain barrier (BBB), but effective drug concentration needs to reach the brain for the therapeutic action. Therefore, developing safe and effective strategies to enhance drug entry in the brain is required to establish ND's treatment. The microneedle-based drug delivery system is one of the effective non-invasive techniques for drug delivery through the transdermal route. Microneedles are micron-sized drug delivery needles that are self-administrable. It can penetrate through the *stratum corneum* skin layer without hitting pain receptors, allowing the phytobioactive compounds to be released directly into systemic circulation in a controlled manner. With all of the principles mentioned above, this review discusses microneedles as a versatile drug delivery carrier for the phytoactive compounds as a therapeutic potentiating agent for targeting mitochondrial dysfunction for the management of ND.

## 1 INTRODUCTION

Central Nervous System (CNS) is a unique and complex part of the human body coordinating the actions and sensory information through signals transmitted from different body parts *via* nerves known as neurons. Neurons are sensitive and delicate cells that need a constant flow of nutritive agents to work effectively [1]. The neurons and glial cells in the brain are supplied continuously with biofluids that maintain the desirable ionic and nutritive microbiome, delivering the necessary entities, and removing unwanted components. They insulate the brain from physical disruptions through a collection of non-cellular and cellular boundaries that communally shield the neuronal tissues against fluctuations in blood modules [2, 3].

To maintain the high energy requirement for vital neuronal activity and synaptic transmission in the brain, neurons and glial cells depend on the “mitochondria”, which is the powerhouse of cells [4, 5]. The primary role of mitochondria is to synthesize adenosine triphosphate (ATP). It also involved balancing membrane potential and ions for synaptic signaling, balancing neuronal redox homeostasis, interchanging neurotransmitters, and neuronal survival and death [6]. Although mitochondria have unique functioning, mitochondrial dysfunction leads to decreased activity of the electron transport chain (ETC) enzyme, generation of reactive oxygen species, and reduction of mitochondrial DNA and caspase three release. Mitochondrial dysfunction is the leading risk factor in the progression of ND, such as Alzheimer’s disease (AD), Parkinson’s disease (PD), Huntington’s disease (HD), Amyotrophic lateral sclerosis (ALS), and Multiple sclerosis (MS) [7]. Many studies have reported many phytobioactive compounds as therapeutic modulators for targeting mitochondrial dysfunction and slowing down the emergence of ND [8]. Clinical care for these diseases is complicated because the BBB acts as a roadblock between extracellular fluid and neuronal tissues, preventing and restricting the influx of harmful substances, xenobiotics, and immune cells within the CNS [9]. The unique and defensive mechanisms of BBB enable the molecules with ionic interaction to cross the barrier for regulating neuronal cells. This ability of BBB is hindered in the case of ND, where pathological changes alter the mechanical rigidity of the BBB. These boundaries restrict the delivery of active pharmaceutical ingredients within the brain, making it challenging to manage neurodegenerative diseases [10]. To overcome these challenges concerning BBB, different techniques are developed. The MN-based drug delivery system is one of the effective non-invasive techniques for drug delivery through the transdermal route. MNs are the novel drug carrier system, micron-sized needles with sub-millimeter-sized devices that are self-administrable and can penetrate through the *stratum corneum* (skin barrier) to improve the drug efficacy, skip first-pass metabolism and avoid gastric irritation with controlled release of drug [11, 12]. As a novel strategy for the carrier of phytobioactive compounds, which will improve mitochondrial functioning, MNs will be a promising approach for developing a practical treatment approach for ND. This review focuses on the role of MNs as a novel drug carrier system for phytoactive compounds targeting mitochondrial dysfunction in ND.

## 2 NEURODEGENERATIVE DISEASES

The nervous system is classified into two; Central Nervous System (CNS) and Peripheral Nervous System (PNS). The CNS comprises the brain and spinal cord, whereas the PNS connects the CNS with receptors and effectors. Neurons are the nervous system's basic units, and the human brain contains billions of neurons arranged in a well-organized network for communication and processing of information to and from body parts of the brain. The components of a neuron are the soma cell body that consists of a nucleus, a slender projection axon, and a myelin sheath insulating around the nerves [13]. Any damage to these neurons is not reversible and is permanent damage that initiates ND. The slow degeneration of neurons of the CNS reduces normal brain functions such as memory, movement, and cognitive activity. These specific brain activities are mainly associated with different regions of the CNS where the neurons are damaged. ND constitutes pathologically modified proteins in the neuronal cells, glial cells, or extracellular spaces between the cells. They also performed specific programmed cell death processes, including apoptosis and autophagy (Table **1**) [14].

### 2.1 Alzheimer’s Disease

Alzheimer’s disease (AD) is a progressive neurodegenerative disease that causes brain cells to degenerate and die. It is the common cause of dementia developed on malfunctioning cortical neurons. Most patients with Alzheimer's disease are older adults, in their 30s or 40s [15] and the disease is associated with memory loss, progressive deterioration in language function, lack of sensitivity, a steady decrease in everyday living skills, and unusual personality and behavioral changes [16]. AD’s psychological and behavioral signs include apathy, delusions, anger, dysphoria, anxiety, abnormal motor behavior, agitation, disinhibition, and hallucinations [17]. Although the specific cause of AD is unknown, the pathological hallmarks of the disease include extracellular and intracellular accumulation of senile plaques and neurofibrillary tangles (NFTs). Senile plaques are the extracellular deposited β-amyloid fragments originating from Amyloid precursor protein (APP), whereas NFTs are the hyper-phosphorylated tau protein from the microtubules of neurons. The protein deposition causes microglia to secrete pro-inflammatory chemicals, which cause neuronal mitochondria to produce more reactive oxygen species (ROS), resulting in oxidative stress and, eventually, neuronal death [18]. It has been proved that mitochondrial dysfunction is mainly involved in AD, associated with loss of mitochondrial biogenesis, oxidative stress-induced respiratory chain dysfunction, mtDNA mutations, and impaired mitochondrial dynamics [19].

### 2.2 Parkinson’s Disease

Parkinson’s disease (PD) is a clinical condition where the motor neurons fail to coordinate action due to the degeneration of neurons at the substantia nigra, a brain region responsible for producing dopamine neurotransmitters. This progressive loss of dopaminergic neurons results in movement-related disorders and other behavioral and psychological symptoms. The clinical manifestation of PD includes asymmetric resting tremors, rigidity, constipation, depression, and bradykinesia. Symptoms such as autonomic dysfunction, pain, and cognitive impairment are the clinical hallmarks of the disease's late stages [20]. The exact mechanism for the disease progression is misery, where degeneration of dopaminergic neurons is the prime cause of motor dysfunction. It is believed that deposition of Lewy bodies containing α-synuclein protein is the pathological hallmark of PD. Increased alpha-synuclein accumulation causes neurotoxicity, further developed due to cellular transmission. Accumulated alpha-synuclein synergizes the pathological event of a synucleinopathy disease that invades the neuronal cells [21, 22]. For targeting PD therapies, the functions of MD and Sirtuins (SIRT1, 2, and 3), Nuclear factor erythroid 2-related factor 2 (Nrf2)-antioxidant response element (ARE) pathway and the mitochondria- endoplasmic reticulum contact sites (MERCs) acts as potential targets [23].

### 2.3 Amyotrophic Lateral Sclerosis

Amyotrophic lateral sclerosis (ALS) is a clinical condition where neurons coordinating voluntary functions fail to execute their effect, leading to difficulty breathing, speaking, and swallowing. It is a progressive neurodegenerative condition caused by the degeneration of neurons in the cerebral motor cortex, brainstem, and spinal cord. Defects in protein production regulation, hyperactivation of microglia, decreased energy supply from reduced MCT (monocarboxylate transporter) 1 transporter, excitotoxicity, cytoskeletal deficiencies, and RNA synthesis disorders such as splicing, miRNA biogenesis, and translation are all pathophysiological processes involved in ALS [24]. ALS occurs by gene mutation of motor neurons identified as Cu/Zn superoxide dismutase one gene (SOD1), TAR DNA-binding protein 43 (TDP43), fused in sarcoma (FUS)/translocated in sarcoma, and ubiquitin 2. SOD 1 gene mutation is the standard form of ALS. The mutant form of SOD present in cytoplasm and mitochondria alters the cell’s normal function, leading to an increase in ROS production, which is the prime cause of apoptosis. Besides that, several cascades of events like neuro-inflammation, glutamate excitotoxicity, protein, and neurofilament aggregations affect neuronal cell normal function, leading to neuronal cell degeneration [25, 26]. Many studies show therapeutic targeting pathways for the management of ALS, such as antioxidative stress, anti-inflammation, metabolism, and mitophagy/degradation [27].

### 2.4 Multiple Sclerosis

Multiple sclerosis (MS) is an inflammatory and demyelinating disorder of the CNS, commonly affecting the youth. It is one of the common causes of a neurological disability, represented by symptoms that involve weakness, weariness, twitches, cognitive difficulties, mood or emotional disabilities, dizziness, convulsion, issues with vision, *etc*. Emotional distress is one of the common symptoms associated with depression representing fifty percent of people with MS. This affects the standard of living and increases anxiety, tension, mental illness, treatment adherence, and disability development [28]. In disease pathogenesis, localized immune cell and cytokines infiltration cause inflammation of white and grey matter tissues in the CNS [29]. It is well understood that MS originated from the peripheral immune response during the initial phase, which is the dominant progressive state of the disease within the CNS [30]. Targeting the metabolism of innate immune cells may be a beneficial therapy for the improvement of disease conditions [31].

### 2.5 Huntington’s Disease

Huntington’s disease is an ND induced by expanded cytosine, adenine, and guanine (CAG) repeats in the huntingtin gene. It is an inherited disease characterized by progressive cognitive decline with psychiatric and behavioral symptoms. Depression, irritability, obsessive, compulsive symptoms, apathy, and psychosis are also seen. Psychiatric symptoms frequently precede the onset of motor symptoms. Schizophrenia (SCZ), bipolar disorder (BPD), major depressive disorder (MDD), and attention deficit hyperactivity disorder (ADHD) are all linked to common genetic variations [32]. The above symptoms of HD were initiated by the expansion of CAG trinucleotide gene expression of the huntingtin gene, which on lysis at the N terminal, results in the formation of aggregates of a polynucleotide. The polynucleotide sequence crosses the neuronal membrane and triggers the pathogenesis of HD, resulting in neuronal death [33, 34]. Active targets like proliferator-activated receptor-gamma coactivator-1alpha (PGC-1α) and regulation of reactive oxygen species (ROS) generation, intracellular calcium homeostasis, and antioxidant activity may be prominent in the management of HD [35].

### 2.6 Multiple System Atrophy

Multiple system atrophy (MSA) is an ND characterized by various symptoms such as autonomic dysfunction, parkinsonism, and ataxia. Slow motion, convulsion, impaired balance, bladder problems, and impaired blood pressure regulation are the common symptoms of the disease [36]. The accumulated aggregates of α-synuclein in oligodendrocytes forming glial cytoplasmic inclusions (GCIs) are the pathological hallmark of MSA. Synucleinopathy is a pathologic process frequently linked to Parkinson's disease and dementia with Lewy bodies. The monomeric and oligomeric synuclein in the extracellular deposits is transferred from neurons, astrocytes, and brain oligodendrocytes and is considered the critical factor for the disease progression. This will significantly induce neurodegeneration, followed by pathological symptoms associated with MSA [37]. Blocking of α-syn arrival to oligodendrocytes, inhibition of α-syn aggregation in oligodendrocytes, inhibition of glutamate-induced excitotoxicity, enhancement of neuroprotection, and inhibition of neuroinflammatory response could be a therapeutic target for the MSA [38].

The diseases mentioned above have varying diagnostic features where a specific domain of CNS degenerates. Before neurodegeneration, pathological breakdown of BBB is initiated, which includes endothelial degeneration, upregulation of luminal adhesion molecules, increased endothelial transcytosis, altered drug transporter expression, perivascular accumulation of toxic product, pericyte degeneration, immune response, and inflammation [39]. Apart from that, the expression of molecules at the tight junction decreases with increased endothelial transcytosis and altered drug transporter expression.

## 3 MITOCHONDRIAL DYSFUNCTION ASSOCIATED WITH ND

ND is a widely affected disease condition globally, with heterogeneous etiologies leading to its progression. The progression of ND is aided by aging, oxidative stress, and mitochondrial dysfunction, commonly associated factors [40]. The human brain requires nearly 20% of total oxygen to function the neurons properly. Most of the neuronal activities are carried out at the expense of ATP produced in a large amount by the mitochondria as an end product of the oxidative pathway. Mitochondrial dysfunction alters this mechanism by decreasing the ATP synthesis, which causes Ca^2+^ imbalance and caspase 3 activations. The imbalance of cellular mechanisms releases more ROS, degrading the outer membrane of mitochondria. ROS are the free radicals capable of producing oxidative stress inside the cells leading to the degradation of the cellular membrane. The mitochondrial inner membrane has a respiratory chain, the mitochondria's structural and functional part. The mitochondrial membrane comprises complexes I, II, III, IV, and V that can catalyze the phosphorylation of ADP to ATP by transferring electrons between the complexes. Any alteration in the functioning of these complexes will affect the synthesis of ATP molecules, followed by mitochondrial membrane disruption due to the secretion of more ROS. The significant release of ROS was initiated with an increase in pro-apoptotic proteins. Similarly, Ca^2+^ ion imbalance causes loss of mitochondrial membrane strength, increasing the pro-apoptotic protein levels within the mitochondria. To minimize the effect of pro-apoptotic proteins, antiapoptotic proteins are also released that bind and hinder the impact of pro-apoptotic proteins [41, 42].

Another mechanism that induces mitochondrial dysfunction is altering mitochondrial DNA (mtDNA). Alteration in genes induces several cascades or events in cellular pathology. Mutation in the gene produces mutant proteins responsible for ROS synthesis in the cytoplasm of the neuronal cell. The ROS are released by the mitochondrial membrane complex III into the cytoplasm of a neuronal cell, causing oxidative stress inside the cell and leading to the death of the neurons [43].

The electron transport chain of the mitochondrial membrane complexes synthesizes ATP on a larger scale during the normal pathological processes. The malfunction of mitochondrial complexes releases the intercellular component cytochrome c. The cytochrome c activates the adaptor molecule apoptosis-protease activating factor 1 (Apaf-1), which generates the apoptosome complex. These apoptosomes activate the caspase 3 and 9 factor that mediates apoptosis’s biochemical and morphological features [44]. Fig. (**1**) shows the mechanism involved in mitochondrial dysfunction.

### 3.1 Mitochondrial Dysfunction in AD

The abnormal activity of mitochondria in AD reduces the energy-releasing processes in the Krebs cycle and glycolysis pathway. The mitochondrial oxidative phosphorylation process is the primary source of ATP synthesis. The process occurs in the electron transport chain from complex I to V using coenzymes to form molecular oxygen, reduced to water [40]. Mitochondria is the largest oxygen consumer in the electron transport chain, generating high superoxide ions. These free radical superoxide ions are ROS, utilized by the antioxidants released by mitochondria as a defense mechanism. The imbalance release of ROS initiates the apoptotic pathway by oxidizing the cellular component that leads to cell death. It was reported that Aβ is a crucial factor in releasing free radicals that activates the cascade of events leading to AD [45].

### 3.2 Mitochondrial Dysfunction in PD

PD is strongly associated with mitochondrial dysfunction’s cascades of events. Superoxide ions are formed during the transfer of electrons from complex I of the electron transport chain. These superoxide ions are released at low levels, which are removed by mitochondrial antioxidants such as manganese superoxide dismutase (MnSOD or SOD2), glutathione (GSH), and peroxiredoxins. Superoxide is converted into hydrogen peroxide and further transformed into water by GSH. The decrease in the level of GSH in the substantia nigra region of the brain is the primary event for PD [23, 46]. The initial recognition of mitochondrial dysfunction as an early event for PD was initiated when MPP+, a metabolite of MPTP (1-methyl-4-phenyl-1,2,3,6-tetrahydropyridine), inhibits the complex I of the electron transport chain and induces PD. A similar kind of study was proposed in the case of rotenone as a mitochondrial complex I inhibitor, which was performed on an animal model of PD, pathologically showing the degeneration of neurons at the substantia nigra [40].

### 3.3 Mitochondrial Dysfunction in ALS

Although the origin of ALS is still not entirely known, mitochondrial dysfunction has been recognized as a primary symptom of the disease. The impaired mitochondrial function arises before the onset of diseases, as shown in *in-vivo* studies of the ALS disease model, by affecting mitochondrial transport, oxidative phosphorylation, and calcium buffering [47].

Many ALS disease model studies provided evidence for the relationship of mitochondrial dysfunction with the pathogenesis of neurodegeneration. In studies carried out using cultured cells and transgenic mice with mutant SOD1, the increased level of a protein involved in mitochondrial fission and decreased protein involved in mitochondrial fusion were observed [48]. In another study, mitochondrial dysfunction is also linked to ALS gene C9orf72 mutations. Motor neurons generated from C9orf72 patients showed a marked reduction in mitochondrial membrane potential [49].

### 3.4 Mitochondrial Dysfunction in MS

Mitochondria provide essential functions in cellular signaling, calcium homeostasis, cell growth, and apoptosis. In many research studies, it has been found that mitochondrial dysfunction is involved in MS pathology. Mitochondrial dysfunction within axons in MS may cause a change in calcium-mediated cytoskeleton and conduction block [50]. Also, it shows abnormal functions of mitochondrial protein and alteration in the mitochondrial DNA, increased oxidative stress, and free radical formation [51].

Chronic neuro-inflammatory stimuli of MS alter neuro-axonal hemostasis, increasing oxidative stress and causing secondary impairment in mitochondria and macromolecules. In neurons and oligodendrocytes, excitotoxicity and an imbalance of neurotrophic substances weaken mitochondrial function and cause increased ROS production. As a result, energy production becomes less efficient, leading to an imbalance between energy production and consumption. This creates an environment that provides insufficient energy levels to demyelinated axons. Because of reduced ATP production, the activation of apoptotic mechanisms occurs through an imbalance in ionic homeostasis [52].

### 3.5 Mitochondrial Dysfunction in HD

The cause of Huntington's disease is the repeat expansion of CAG in the mutant HTT gene, which leads to polyglutamine tract enlargement in the N‐terminal of the Huntington (Htt) protein. This creates aggregation of mutant Htt protein with expanded polyglutamine. This aggregation brings in additional proteins and mitochondria, interfering with the transportation of mitochondria along dendrites and axons and severely impacting mitochondrial fission-fusion [53]. Also, mutant Htt can directly interact with the mitochondrial membrane to disrupt the membrane and enhance the Ca^2+^ and additional apoptosis inducer sensitivity [54].

Mitochondrial dysfunction also causes due to increased ROS production in both HD patients and mice models [55]. ROS causes protein misfolding and inclusion bodies formation. These inclusion bodies assemble axons and dendrites, including neurons, and inhibit the transmission of neurotransmitters. The biochemical analysis of the brains of HD patients, transgenic mice, and other HD models showed decreased enzyme activities, proving the role of mitochondrial dysfunction in HD pathogenesis [56, 57].

### 3.6 Mitochondrial Dysfunction in MSA

MSA is a neurodegenerative condition that involves the accumulation of α-synuclein in oligodendrocytes forming glial cytoplasmic inclusions (GCIs). Mutated COQ2 is an enzyme required for biosynthesis coenzyme Q10 (CoQ10) involved in MSA. In mitochondrial electron transport chain (ETC) and antioxidant, CoQ10 transports electrons. In MSA, brain tissue impairment of CoQ10 has proved and thus the association with mitochondrial dysfunction in MSA. The study has been carried out to investigate mitochondrial dysfunction in MSA by observing ETC activity in control and MSA brain tissue. The activities of occipital and white matter ETC complex I, II/III, and IV were measured and showed increased activity of I and IV complex with decreased activity of II/III complex. This is consistent with a deficiency in CoQ10 as discussed in the MSA condition and demonstrates a high regional pathogenic load of GCIs [58].

## 4 TREATMENTS FOR NEURODEGENERATIVE DISEASES

Many factors are involved in neurodegenerative diseases, such as molecular, cellular and genetic factors. These factors exert their effects early in a neurodegenerative disease patient’s life. The available treatments do not target all sites or disease conditions but reduce the patient’s symptoms from getting worse. Various other therapies available for neurodegenerative diseases are discussed below.

Recently, many ND therapy-related studies have focused on protein misfolding. These include preventing the development of disease-related proteins, preventing their accumulation, limiting their distribution, and reducing their toxic effects. With this goal, chemical agents that can restrict the accumulation of aberrant proteins, chemical modulators of autophagy, or specific antibodies that remove any misfolded proteins are developed for ND treatment [59]. Drugs like amantadine are used to treat Parkinson's disease as a mild glutamate receptor antagonist, which increases dopamine and decreases dopamine reuptake. Amyotrophic lateral sclerosis is treated with the tyrosine kinase inhibitor masitinib. According to certain *in-vitro* and *in-vivo* experimental models, the aberrant glial cells that proliferate in amyotrophic lateral sclerosis may be sensitive to these inhibitors. Galantamine is a marketed drug for AD treatment. It can facilitate neuronal impulses, cross the blood-brain barrier, and hinder acetylcholinesterase in the brain [60]. Nanoformulation plays a vital role in overcoming the disadvantages shown by conventional therapies. The nanoformulations used to treat various types of ND are solid lipid nanoparticles, liposomes, nanoemulsions, nanostructured lipid carriers, carbon nanotubes, and PLGA nanoparticles [61]. Stem cells are also used in the management of neurodegenerative diseases. These cells can continuously regenerate themselves and differentiate into almost every other form of cell. Mesenchymal stem cells are more acceptable since they are readily available in sufficient numbers and have low immunogenicity. Also, they have been the subject of numerous investigations and clinical trials for neurodegenerative diseases [62]. However, current treatments are effective in treating ND, but repeat dose requirements and crossing BBB limit their applications in the management of ND. Therefore, finding novel therapies for managing ND with fewer adverse effects is of great importance.

## 5 ROLE OF BBB IN BRAIN DRUG DELIVERY

The BBB is a semipermeable membrane between blood and the brain. It is composed of endothelial cells tightly bound to each other, preventing the transfer of solutes from blood to CNS extracellular fluid where the neurons reside and providing necessary nutrients for the proper functioning of the brain cells [63]. BBB consists of endothelial cells lining the brain’s capillaries bounded by astrocytes and pericytes. The astrocytic end-feet processes form the central portion of the BBB. They are not involved in the barrier function of the brain. However, they are responsible for producing an atrophic factor essential for the functioning of BBB endothelial cells. These cells consist of complicated tight junctions between the endothelial cells composed of multiple transmembrane proteins and have an enhanced mitochondrial element with no perforations, limited pinocytotic function, and minimal pinocytotic activity. This potentially locks the paracellular process and differentiates the endothelial cell membranes into dual layers: luminal (blood side) and abluminal (brain side). Pericytes are bounded in an irregular interval to the abluminal membrane of endothelium cells. The void spaces between the endothelium pericyte and the basement membrane-filled astrocyte foot network form the blood and brain interface [64-67]. The BBB maintains the internal brain condition by keeping the cerebrospinal fluid (CSF) and brain interstitial fluid (BIF) composition below the acceptable margin. Thus, the neurons could operate the complicated integrative activity [68]. Neuroprotection is a significant feature of the BBB, especially from toxic substances. It also safeguards the brain. The boundary serves to distinguish the neurotransmitters that function centrally and peripherally. Subsequently, the continuous recycling and clearing of CSF and BIF by excess outflow enable clarifying bigger particles and brain metabolites to retain the brain microenvironment [69].

To cross the barrier, a drug should be small, unionized, and lipid-soluble. It should be of small molecular size and have a good partition coefficient. It should have systemic enzyme stability, plasma protein binding affinity, uptake into other tissues, clearance rate, and effects on existing pathological conditions [70]. There are four main mechanisms through which the substances can cross the BBB. They are:

• Paracellular Diffusion
It refers to transporting substances across the membrane by passing through the intercellular spaces between the cells. The mere existence of close junctions in endothelial brain cells significantly restricts paracellular diffusion. Tiny molecules soluble in water can diffuse BBB by penetrating rigid boundaries. It is in contrast to transcellular transport, where the transportation of substances is performed by a cell through a cell. This mechanism can transport substances with high lipid solubility and lower molecular weight.
• Carrier-mediated Transport
It allows substances like glucose and amino acid to cross the membrane barrier by binding with a carrier protein. This process leads to temporary, small openings caused by linking the specific substratum to the carrier, enabling substratum molecules' movement. In this transport mechanism, only specific carriers can be used to transport the nutrient molecules across the membrane barrier.
• Receptor-mediated Endocytosis
It is a process by which cells absorb metabolites, proteins, and hormones by invagination with the help of receptors available at the barrier’s luminal side. Lipophilic substances are interiorized in the brain as low-density lipoproteins (LDLs), identified by endothelial cells, and endocytosed. Polycationic proteins can pass through the BBB *via* absorptive transcytosis regardless of specific plasma-membrane receptors. Here, endocytosis involves interaction between polycationic compounds with the negative charges on the endothelial cell [71].
BBB restricts the delivery of active pharmaceutical ingredients within the brain, making it challenging to treat ND. While the BBB becomes weakened and is more penetrable in many ND conditions (*i.e*., Alzheimer’s disease, Parkinson’s disease), the delivery of therapeutic agents into the brain can still face critical challenges. The main reasons for the failure of brain delivery of drugs are poor penetration of the drug across the BBB and transport of drugs back (efflux) from the brain to the blood.

### 5.1 Strategies to Bypass the BBB

BBB provides limited permeability and obstructs the delivery of neuroprotective drugs. These hurdles of the BBB pose difficulties in diagnosing and treating ND. Hence, several strategies were developed to enhance the delivery of drugs across the BBB, including invasive and non-invasive techniques. Invasive techniques directly deliver the drug into the brain *via* intracerebroventricular infusions, polymer or microchip systems, and transient disruption of BBB. In contrast, non-invasive techniques modify the molecules through medicinal chemistry techniques for active targeting, improve the permeability using nanocarrier, and utilize new routes for targeting the brain *via* an intranasal drug delivery system [72].

#### 5.1.1 Invasive Techniques

Invasive techniques involve direct drug delivery into the brain *via* the spinal canal to reach cerebrospinal fluid using injectables, implants, and biological tissues through intrathecal, intracerebral, and intraventricular routes [73]. The intracerebral implant is another device that delivers biocompatible polymer-coated drugs into the brain. The polymer degrades biologically to release drugs within the targeted brain region over an extended period [74, 75]. Temporary breakdown of BBB provides direct access to blood components with brain cells. Various methods initiate this, including using vasoactive drugs such as bradykinin and physical methods for stimulation such as ultrasound [76]. Furukawa *et al.* investigated the effects of subthalamic nucleus deep brain stimulation surgery on drawing skills and cerebral perfusion in PD patients. Deep brain stimulation of the subthalamic nucleus was created to help patients with PD [77]. The suggested application of cervical spinal cord stimulation to treat and prevent cognitive decline in dementia and ND was investigated by Tomycz *et al*. Stimulation of the cervical spinal cord may increase cerebral blood flow and, therefore, helps in better healing. They suggested cervical spinal cord stimulation as a titrable, programmable, extracranial neuromodulation strategy to improve blood flow and cognitive function and prevent cognitive impairment in dementia and ND [78].

#### 5.1.2 Non-invasive Techniques

Non-invasive techniques modify the drug molecules to enhance their properties, like permeability and solubility, by chemical alteration. These approaches improve the brain-targeted delivery of drugs, such as prodrugs, in the most advanced manner. Prodrugs are drug molecules chemically modified in an inactive form that crosses the BBB and metabolizes to form an active drug within the brain in a single-step process [79]. Levodopa has a normalizing impact on default-mode network connection in non-demented PD. According to Zhong *et al.,* PD is characterized by nigral degeneration, which causes a drop in brain dopamine levels. Even in people with no significant cognitive dysfunction, it is well known that the default mode network (DMN) characteristic is disturbed in PD. Levodopa is the dopamine prodrug, and its therapy showed decreased DMN connectivity in PD patients [80].

The chemical method involves the conversion of a biologically inactive molecule to an active therapeutic agent by various chemical reactions. It is designed by recognizing specific enzymes at the target site, providing entry into the brain by enhancing the lipid solubility of the molecule. The lipidic moiety is susceptible to rapid chemical reactions such as oxidation, leading to a charged and highly polarized intermediary residue that restricts the compound from re-diffusing from the BBB [81]. Intranasal delivery is an efficient route for drug administration. The drug is directed from the nasal cavity to the brain, bypassing the BBB. This method has many drawbacks where it allows only a small volume of drugs to get administered through this route. Enzymatic drug degradation and limited area for drug absorption are other limitations associated with the intranasal route [82]. The BBB surface has a variety of receptors, including those for proteins, peptides, and antibodies. These compounds act as surface-active ligands, assisting translocation *via* receptor-mediated transcytosis. At the same time, the cationic vehicle passes across the BBB *via* absorption-mediated transcytosis. Another method is vehicle-mediated transcytosis, which involves the utilization of additional nutrients like glucose and glutathione, which attach to the vehicle surface and aid in its translocation [83]. MN-based drug delivery systems are the novel emerging non-invasive technique for delivering phytobioactive compounds.

## 6 MICRONEEDLES (MNs)

MN are micron-sized needles with a sub-millimeter size; from these needles, drugs can be transferred across the skin without discomfort. It overcomes the drawbacks of the traditional method of drug delivery. The oral route hinders the absorption of most drugs due to the acidic nature and enzymatic actions in the gastrointestinal tract. The alternative to the oral way is a hypodermic injection. Though there is 100% bioavailability, the patients do not prefer it since it is a painful procedure and requires a skilled person for administration. The drug delivery through the transdermal route is hindered due to the presence of a primary barrier: *stratum corneum*, a layer of the skin made of dead corneocytes. This would significantly reduce the efficiency of drug distribution and restrict the types of drugs transported *via* the transdermal drug delivery system (TDDS). MN is an advanced technology that allows macromolecules to reach various skin layers. Drug delivery employing an MN device enables pharmaceutically active agents to pass through the *stratum corneum*, causing an adequate amount to penetrate the tissue. Besides enhanced therapeutic benefits, MN offers highly reliable reproducible findings with limited inter-subject variation in bioavailability [84-86].

### 6.1 Properties of MNs

MN micrometer-sized needle structures that adhere to the flat substrate. It has a quick onset of action since the drug enters systemic circulation. The needles do not touch the pain receptor; therefore, it is painless therapy, and self-administration of the patch is possible, which offers better patient compliance. The MN increases the drug bioavailability as it bypasses first-pass metabolism and conjugation reactions. It enhances the drug permeability due to the mechanical property of the needles to pierce the SC layer and deliver the drug to the dermis layer [87].

### 6.2 Drug Delivery Mechanism

Various diffusion mechanism accompanies the therapeutically active agent administration dermally. The administration of phytobioactive agents temporarily distorts skin by using microneedle technology. A microneedle system is designed by placing hundreds of micron-sized needles in arrays on a small patch to deliver enough medication to provide the necessary therapeutic response. The needles create micron-sized pores in the outermost layer and deliver the loaded drugs into the layers of the skin without causing any damage to the underlying tissues. The medication is inserted directly into the outermost layer of the skin or the superficial dermis that later enters the bloodstream, displaying pharmacological action when it reaches the target [85, 88].

### 6.3 Design and Structure of MNs

MN can be of different shapes and dimensions based on what kind of delivery is required. The MN structure can be solid, hollow, dissolving, and coated. The tips of the MN can be tubular, trilateral, tapering, pentamerous, octal, and modified in several other forms. MNs are designed in various sizes based on the MN type and the materials available for their fabrication. Since the epidermis depth is close to 1500 μm, the MN can be close to 1500 μm for successfully delivering therapeutic agents *via* the epidermis. If length is further increased, the needles enter deeper layers into the dermis, causing nerve impairment and pain. They usually have a length of 150-1500 μm, a width of 50-250 μm, and a 1-25 μm thickness [88, 89]. MN is manufactured by using various components. The variety of materials used in MN manufacturing is metals and polymers. All resources used to manufacture MNs should meet the essential characteristics such as durability, economical, user-friendly, non-erodible, and low physical tolerance [90].

### 6.4 Materials and Methods Used for Fabrication of MNs

#### 6.4.1 Materials Used for Fabrication of MNs

##### 6.4.1.1 Silicon

The MNs were first fabricated using silicon. Due to its malleable nature, needles with various dimensions and types can be fabricated. MNs made from silicone have tremendous mechanical power to penetrate the skin. Short silicon MNs with a chromium mask were manufactured using the silicon dry-etching method involving reactive ion etching. Silicon plays a significant part in manufacturing micron-size structures and microelectromechanical devices (MEMS). Since MN fabrication using silicon is lengthy, costly, biologically unacceptable, and breaks easily, its use is limited [91, 92].

##### 6.4.1.2 Metal

Metals like stainless steel, nickel, palladium, and titanium were used to manufacture MN. Metals exhibit excellent biological acceptability, which can also reach the various layers of the skin without any damage. Metals are stronger and less brittle than silicon. Therefore, metals are pretty comfortable while processing MN [84, 93].

##### 6.4.1.3 Ceramic

Ceramics like alumina, gypsum, brushite, and a few modified ones are used. Alumina (Al_2_O_3_) is primarily used for its resistance to chemicals. This creates solid oxide because of the intensely powerful electrovalent and covalent links between aluminum and oxygen atoms. Currently, ormocers are used, obtained by organic modification of ceramics. A polymer of various characteristics may indeed be developed utilizing different organic elements through polymerization. It is manufactured primarily employing a micromolding method. Ceramic sludge is moulded using micromoulds since micromolding ways tend to be more accessible and provide scope for leveling up [94, 95].

##### 6.4.1.4 Silica Glass

Silica glass can be used to prepare MNs of varying sizes and shapes. The use of glass will generate varying geometries on a small scale. Silica glass is physiologically inert but fragile. Borosilicate glass containing silica as well as boron trioxide is very stretchy. They break easily and hence are not preferred in the fabrication of MN [96].

##### 6.4.1.5 Carbohydrates

Carbohydrates like maltose, osmitrol, trehalose, saccharose, xylite, milk sugar, and glycans can be incorporated in MN fabrication. They are cost-effective and physiologically compatible. The slurries of carbohydrates are moulded using silicone or metal templates. The obtained slurry is poured into the microneedle molds to form MN. Time-based carbohydrate dissolution controls drug release within the skin but designing an MN using these carbohydrates is not easy as it deteriorates under increased temperature [97].

##### 6.4.1.6 Polymers

Polymer-based MNs are used due to their outstanding biocompatibility, biodegradability, and nontoxicity. The various polymers like polyglycolic acid (PGA), poly (lactic-co-glycolic acid) (PLGA), poly (methyl vinyl ether-comaleic anhydride), poly (methyl methacrylate), poly (vinylpyrrolidone) (PVP), polylactic acid (PLA), cyclic olefin copolymer, polystyrene (PS), and poly (vinyl alcohol) (PVA) are used. Dissolving MNs are prepared by using these polymers [98, 99].

#### 6.4.2 Methods of Manufacturing MNs

Various techniques can be used to develop needles with multiple heights and tip angles.

Solid MN is fabricated by anisotropic wet etching, Silicon dry-etching process, isotropic etching, and three-dimensional laser ablation. Metal MNs are developed by metal electroplating, laser cutting, and wet etching methods. Coated MN was produced by coating the MN using the solution containing the surface-active agents, the drug, and a stabilizer in water with enhanced viscosity to keep more formulation while drying. MNs are immersed in the liquid mixture containing drug and other additives several times until the required thickness is obtained. All the needles in the patch can be immersed in the microwell with drug solution; otherwise, a drug layer developed in advance using a roller can be mounted on the needles, known as the layer-by-layer coating method. Micro moulding technique is used in the fabrication of dissolving MN. Hollow MNs are prepared using integrated lithographic moulding technique, Micro-electromechanical systems techniques like laser micromachining, micro-fabrication, wet chemical etching, and deep X-ray photolithography [100].

### 6.5 Types of MNs

MN can be designed in forms like solid, dissolving, coated, hydrogel-forming, and hollow MN. These designs show differences in the delivery of drugs since few of these are designed to develop minute holes in the outermost part of the epidermis, few with a layer of drug masking the outer layer, few which get solubilized in interstitial fluid, and few containing the drugs at the core [101].

#### 6.5.1 Solid MNs

Solid MN is designed to develop minute holes in the outermost layer of the skin. The pointed front part of MN pierces the different layers of the skin, creating minute holes. Thus, when the formulation is applied on the skin surface pierced with the solid MN, it quickly permeates through the various skin layers and reaches systemic circulation, showing the quick onset of action. Drug delivery is mainly followed by passive diffusion. After drug delivery, the microchannels must be closed to avoid entering toxic substances. Usually, solid MNs are made of silicon, polymers, and metals [102]. Developed gold-coated solid silicon MN found to have 250 μm in height, 52.8 μm base width, 4.73 aspect ratio along with 24.5° tip angle and 45 μm diameter. The studies showed a greater absorption rate and physical resistance in the case of solid MN [103]. Song *et al.* investigated the delivery of cimetidine by the transdermal route through microneedle-treated skin and checked the effect of drug ionization on the permeation. Cimetidine is a water-soluble drug and has poor skin permeability. So here, they attempted to deliver the drug through the skin, pretreated with maltose MN. Skin is first pierced with MN, then the permeation rate of cimetidine through the pretreated skin in the form of gel was evaluated. It was found that the penetration rate of the drug was better [104]. Ilic *et al.* investigated the combined effect of solid MN of biocompatible and nanoemulsions to enhance drug transport through the skin and carried out *in-vitro* and *in-vivo* studies. In this study, the skin was pretreated by using MN. This resulted in better bioavailability of the drug, and also, the drug amount retained in the skin was increased [105].

#### 6.5.2 Coated MNs

In this, the drug is coated onto the surface of the MN. When the microneedle patch is applied, the drug layer on the surface of the needle starts dissolving in the interstitial fluid and thus shows the systemic effect. It is a preferred method for delivering drugs with high molecular weight for rapid drug delivery. Since the coated drug will be in a solid form, it shows long-term stability. The method used for coating MN is dip coating, which involves dipping the MN into the solution of the drug and then withdrawing. As a result, a continuous liquid film adheres and dries on, leaving behind a uniform coating. The process is continued until the desired thickness of the drug is obtained. Hereafter applying the needle patches to the skin, the formulation is dissolved and released; subsequently, the patch is removed [106, 107]. Jain *et al.* developed coated MN loaded with 5-Aminolevulinic acid (5-ALA). MN was coated with 5-ALA using a dip coater. The MN successfully penetrated the skin and was better than other topical preparations [108].

#### 6.5.3 Dissolving MNs

Dissolving MN has been drawing attention in recent years since these MN get degraded in the body quickly when it comes in contact with the body fluids because they are made up of biodegradable polymers. The micromoulding technique is used for the manufacturing of MN. The drug is entrapped within the polymer. The drug-polymer mixture is poured into the micromoulds to fill the mold's micron-sized cavities under vacuum or pressure and then dried under ambient conditions, centrifugation or pressure. When the microneedle is inserted, it penetrates through the various barriers of the skin, and then the polymer undergoes degradation, thereby drug released into the systemic circulation. The drug release rate depends on the rate of polymer degradation. It can be used for long-term treatment since the polymers are biocompatible. The bio acceptance and degradation of the polymer in the dermal tissue make this an excellent choice to treat chronic conditions with better acceptance by patients. Successful needle drug distribution is an essential aspect that faces challenges when developing dissolving MN. Hence, polymer-drug blending becomes a crucial measure in manufacturing MN. The main advantage of dissolving MN is that it needs not be removed once the needle is inserted since the needle gets dissolved in the body [109, 110].

PVP and poly(methylvinylether/maleic acid) are utilized in MN fabrication. It was found that the needles dissolved easily when inserted into the skin, and the drug release was good. MN was found to have good mechanical strength and permeation through the skin layers [111]. Yao *et al.* undertook a study involving the fabrication of dissolving MN to increase levonorgestrel's transdermal delivery. This is then evaluated by performing *in-vitro* and *in-vivo* analyses. The MN is obtained by the micromoulding method using the polydimethylsiloxane mould. MN was found to have good mechanical strength and penetration properties, and the drug loading capacity of the MN was better than the conventional MN [112].

#### 6.5.4 Hollow MNs

They are fabricated in such a way that they contain an empty void. The space inside the needle is filled with the drug either in the form of solution or dispersion. These MN tips have a hole. When the needles are inserted, they pass through various layers of skin with the simultaneous release of the drug through the opening at the tip of the MN. It is mainly applied for the delivery of molecules having high molecular weight. This type of MN can deliver enormous amounts of the drug because the needle can accommodate more drugs inside the hollow space. But maintaining a constant flow rate is essential. Increasing the microneedle’s bore would maximize its discharge speed and reduce the needle's sharpness. The metal cover is often added to the MN to improve microneedle power; however, it will sharpen the tips [113, 114].

#### 6.5.5 Hydrogel Forming MNs

Recently, MNs are developed using super-swelling polymers with a hydrophilic structure that enables them to absorb a large volume of moisture through the polymeric network. Such materials expand once interstitial fluid comes into contact after being injected into the skin. Hence, minute openings are created between the capillary discharge and the MN device. Such MNs are mainly employed before needling only to break the skin boundary. The polymer matrix act as a rate-limiting matrix upon swelling. These have dimensional and structural versatility. The unique characteristics of these MN include simple sterilization and unaltered extraction from the applied surface [115, 116]. Migdadi *et al*. worked on hydrogel-forming MN for the transdermal administration of metformin to overcome the adverse effects of oral delivery. Findings showed enhanced penetration and the absorption rate of the drug with developed MN [117]. Different types of microneedles are shown in Fig. (**2**).

### 6.6 Methods of Delivering Drugs by MNs

One of the methods through which the drug release through MN occurs is by creating minute holes on the skin surface using the MN. When the formulation is applied on this skin surface pierced with the MN, it quickly permeates through the various layer of skin and thereby reaches the systemic circulation. Iontophoresis may further speed up drug penetration through the skin. Drugs can also be coated onto the surface of the MN, where the drug layer on the surface of the needle starts dissolving in the interstitial fluid of the skin and thus shows the systemic effect. Otherwise, the MN can be dipped into the solution of the drug and then inserted into the skin's surface. The other method is to encapsulate the drug within the biocompatible polymer. This drug-polymer mixture is then, by the micromouding technique converted to MN. Another approach is to build a hollow MN, so that medicament in the form of solution could be poured into the MN cavity [118].

### 6.7 Patient Compliance

The MN penetrates the *stratum corneum* layer, overcoming the skin barrier function without piercing the deeper layer of the skin where the pain receptors are located and hence responsible for the delivery of the drug without causing any pain, unlike other dermal injections. Furthermore, the pain severity relies upon the number of MN in a patch, the size of the microneedle, and the type of needle. Reducing the size and number of the MN on the patch minimizes the discomfort involved during the treatment [101, 119-121]. The perceived benefits of microneedles included the potential for self-administration and decreased discomfort, tissue damage, and infection risk [116, 122, 123].

### 6.8 Advantages of MNs

The MN can act as an excellent carrier for phytobioactive compounds due to its following advantages. Administration of large molecules is easy, thereby avoiding first-pass metabolism. It gives faster healing in the injection area when compared to hypodermic needles. Also, it provides good patient compliance and is simple to handle. Moreover, it guarantees reduced pathogenic permeation than that of injectables because MNs rupture only the outermost layer of the skin. Applying medication to the specific skin region improves the effectiveness of the therapeutic agent at lower concentrations with excellent tolerability and no swelling or redness for a prolonged period. The quick dispersal of medications was accomplished by integrating the MNs with other techniques [124, 125].

## 7 ROLE OF MNS FOR THE DELIVERY OF PHYTOBIOACTIVE COMPOUNDS IN TARGETING MITOCHONDRIAL DYSFUNCTION FOR THE MANAGEMENT OF ND

In the central nervous system, macrophages play an important role in regenerating neurons, inflammation induction, and angiogenesis. They also manage matrix remodelling for autoimmune diseases, mental disorders, and neurodegenerative diseases. It has been reported that macrophage/monocytes (macrophage receptors) naturally can cross the BBB and move through the brain-ventricular choroid plexus. During tumours and inflammations, CNS parenchyma releases chemokine gradients which could induce macrophages to migrate into the endothelium of BBB and migrate into brain parenchyma. This property of macrophages allows them to deliver drug molecules into the brain. The transdermal delivery by microneedles loaded with drug molecules provides delivery of active ingredients through the stratum corneum and is released in the peripheral circulation. Once the drug molecule releases into the peripheral circulation, macrophages can engulf, process, and deliver them at the site of disease inside the brain [126, 127].

The phytobioactive compounds used in the treatment of ND have severe gastrointestinal adverse effects such as abdominal discomfort, loss of appetite, nausea, and vomiting. Even most phytobioactive compounds lose their active potency by gastrointestinal secretion, like acid, juices, and enzymes, followed by degradation with favored gastrointestinal microorganisms [128]. Therefore, an alternate and more convenient drug delivery strategy has been devised through a transdermal route *via* the MN array. MNs are the modified form of a conventional transdermal patch that has played a promising role in delivering phytobioactive compounds into systemic circulation. MN patch delivers the pharmaceutically active drug inside the skin barrier. Hence, it plays a significant role in the management of ND [129]. MN patch system consists of a microneedle array integrated on a backing layer of circular or square shape. The patch is applied on the skin with minimal pressure for proper penetration of the microneedle into the *stratum corneum*, thereby increasing the drug absorption rate.

As discussed earlier, in many clinical investigations and experiments, the involvement of mitochondria in ND has been reported. Therefore, finding the neuroprotective agent has properties to protect mitochondria in CNS may be a prominent way to develop ND treatment.

The phytobioactive compounds act by various mechanisms on mitochondrial dysfunction in the management of ND. These mechanisms involve the modulation of mitochondrial bioenergetics, mitochondrial biogenesis, mitochondrial membrane potential, modulation of mitochondrial calcium homeostasis, neuroprotection against mitochondrial oxidative stress, and modulation of mitochondrial fusion and fission dynamics [130].

Many phytobioactive compounds such as fisetin, ferulic acid, quercetin, protopanaxadiol, Epigallocatechin-3-gallate (EGCG), α-arbutin, viniferin, auraptene, oleuropein, astaxanthin, nobiletin, geniposide, glycyrrhizic acid, huperzine A, ferulic acid, hydroxytyrosol, curcumin and bacopa moneri are reported as therapeutic modulators for targeting mitochondrial dysfunction in the management of ND (Table **2**).

Alikatte *et al.* proved the neuroprotective activity of fisetin, where they found fisetin may improve the mitochondrial enzyme activity and the pathogenesis of PD [131]. The protective efficacy of ferulic acid against mitochondrial dysfunction was investigated by Anis *et al*. using a 6-hydroxydopamine lesioned rat model of Parkinson’s disease [132]. Dezfouli *et al*. investigated the effect of silent information regulator (SIRT 1) signaling and mitochondrial biogenesis by melatonin neuroprotection using rat models of intra-hippocampal Aβ injection-induced cognitive impairment. This study gave a new strategy by using melatonin to manage AD [133]. Ay *et al*. found a significant effect of quercetin, which improves mitochondrial biogenesis, which was investigated using dopaminergic neuronal models, and further checked its effect in a transgenic mouse model of PD [134]. The neuroprotective effect of protopanaxadiol was investigated by Bak *et al*. in PC 12 cells. This study suggested that the antioxidant effect of the drug was responsible for neuroprotection and enhanced mitochondrial function [135]. Chen *et al*. investigated the ability of Epigallocatechin-3-gallate (EGCG) to recover from cellular injury and mitochondrial dysfunction. They suggested that EGCG is a novel therapeutic agent to target mitochondria in brain disease [136]. Ding *et al*. demonstrated a significant effect of α-arbutin on rotenone-induced mitochondrial dysfunction and apoptosis of human neuroblastoma cells. They found excellent neuroprotective activity in α-arbutin, and it can be used as a potent therapeutic agent for the treatment of PD [137]. Fu *et al*. demonstrated decreased levels of ROS and avoided loss of mitochondrial membrane potential by viniferin in a cell expressing mutant Huntingtin disease [138]. Jang *et al*. investigated the role of auraptene in protecting neurotoxin-induced reduction of mitochondrial respiration and inhibiting ROS generation. This study suggested preventive action of the phytobioactive drugs can help in the improvement of PD-related neurobiological conditions [139]. Kim *et al*. investigated the effect of oleuropein on HT-22 hippocampal neuronal cells against glutamate-induced toxicity. They found that it inhibits the translocation of mitochondrial apoptosis-inducing factors to the cytoplasm of HT-22 cells [140]. The protective effect of astaxanthin on MPTP-induced apoptosis of the substantia nigra neurons in the mouse model of PD was demonstrated by Lee *et al*. From the obtained results, they suggested that astaxanthin can be used as an effective drug in treating ND, such as PD [141]. Nobiletin improves neuron's partial mitochondrial depolarization and decreases mitochondrial calcium overload and ROS generation. With these findings, the need for further study to understand the exact molecular target of nobiletin in mitochondria was suggested by Lee *et al.* [142]. Geniposide significantly affected mitochondrial dysfunction in AAP/PA1 mice by suppressing mitochondrial oxidative damage and increasing the mitochondrial membrane potential and activity of cytochrome C oxidases. Lv *et al*. showed that geniposide could improve memory deficits by decreasing mitochondrial oxidative stress and can act as an effective therapeutic agent in preventing AD progress [143]. The effect of glycyrrhizic acid on mitochondrial dysfunction and biogenesis in aluminum toxicity in the PC 12 cell line was investigated by Rashedinia *et al*. Glycyrrhizic acid significantly enhanced the expression of the mitochondrial gene. Hence, they suggested it can be used as a supplement in the management of ND [144]. Yang *et al*. studied the beneficial effect of Huperzine A on mitochondrial dysfunction and memory deficits in AβPP/PSI double transgenic mice. They described the novel mechanism of neuroprotection provided by Huperzine A and gave an idea for finding a new therapeutic agent for Alzheimer’s disease [145]. Ferulic acid showed improved mitochondrial dysfunction in Alzheimer’s disease on prolonging dietary supplementation, as reported in a study carried out by Zafeer *et al*. using streptozocin-induced sporadic dementia of Alzheimer’s type [146]. Hydroxytyrosol can improve mitochondrial function and reduces oxidative stress potentially through activation of the AMPK pathway in the brain of db/db mice [147]. Many phytobioactive compounds can be formulated as MN patches to manage ND effectively. Tao *et al.* studied the neuroprotective effect of HupA against iron overload-induced injury in neurons. They found that Hup A significantly reduced the iron overload-induced decrease in neuronal cell viability and ROS and increased ATP by targeting mitochondrial dysfunction and oxidative stress [148]. A cellular model of PD using siRNA-mediated impaired PTEN-induced kinase 1 (PINK1) in SH-SY5Y neuroblastoma cells was developed by Merwe *et al*. to check the protective effect of curcumin. The finding showed a significant effect of curcumin by attenuating mitochondrial dysfunction and apoptosis to improve the down-regulation of PINK1 [149]. To understand the neuroprotective effects of the BM against acrolein and H_2_O_2_ and the mechanism involved, Singh *et al.* conducted a study using the SK-N-SH human neuroblastoma cell line. They found the neuroprotective effect of BM through inhibition of intracellular ROS generation, improved mitochondrial functions, and balancing of redox proteins (NF-κB, Sirt1, ERK1/2, and p66Shc) which was impaired in a disease condition [150].

Huperzine A (Hup A) is a Chinese herb effective as an acetylcholinesterase inhibitor and can improve the memory impairment of AD patients. Commercially it is available as tablets, capsules, and intramuscular injections that have specific side effects on the gastrointestinal tract and injection-site adverse reactions. To improve the delivery by minimizing the adverse effects of Hup A, dissolving transdermal MN patches were prepared by Yan *et al.* Transdermal route avoids the side effects of the oral and intramuscular route while still offering sustained drug administration. This can further be improved *via* an MN patch as it can penetrate the skin barrier providing better systemic absorption [151].

Curcumin is known for its neuroprotective property and can minimize the symptoms of PD. Clinically curcumin has limited use due to poor solubility and bioavailability. It was improved by the formulation of MN incorporated with solid lipid nanoparticles (SLN). Prabhu *et al*. have prepared SLN by microemulsion technique by varying the concentration of glyceryl monostearate and tween 80. The optimized formulation of SLN was incorporated in an MN patch fabricated by micromolding technique and evaluated for neuroprotective effect [152]. Bacopa monnieri (BM) is a herbal drug that can improve different nervous disorders, intellect, and memory enhancement. Nanoformulations containing lipids have gained more attention in targeting CNS disorders. With this understanding, Joy *et al.* attempted to incorporate BM-loaded SLN into a microneedle patch to elucidate its neuroprotective effect in managing PD. The quality by design (QbD) approach was used for optimizing the SLN’s formulations. The developed BM-loaded SLN MN patch was mechanically robust and showed nonirritant properties. Decreased degree of bradykinesia, increased balance ability, and motor coordination. This study concluded that the MN technique could enhance the therapeutic efficacy and bioavailability of BM *via* transdermal absorption [153].

The above research work analyzed the faster delivery rate of the drug in comparison with conventional dosage forms. MN penetration capability increases the concentration of the drug within the body without posing fluctuation in the dose of the drug. This is one of the significant applications of the microneedle, which ensures its application in ND management.

Several types of research have been conducted with drug-loaded microneedle, where its role in treating ND is more promising. Zhou *et al.* developed a microneedle patch incorporated with levodopa to treat PD. The matrix of the MN patch contains gelatin-methacryloyl, where levodopa is stored in mesoporous silica-coated upconversion micron-rods sealed by azo molecules. These azo molecules oscillate on exposure to near-infrared radiation, which initiates the controlled release of levodopa from the MN patch. The MN patch showed good biocompatibility with the controlled release of the drug and enhanced levodopa's efficacy in treating PD [154].

Singh *et al.* developed microneedles incorporated with ropinirole hydrochloride delivered *via* iontophoresis to enhance the transdermal controlled delivery of ropinirole hydrochloride. It was compared with an extended-release tablet, showing reduced dose flexibility. To improve the penetration of the drug, the iontophoresis technique was used where electrical impulses were applied to the MN patch. Combined modulated iontophoresis of MN was compared with modulated iontophoresis of ropinirole hydrochloride, where combined modulated iontophoresis of MN showed better action of ropinirole hydrochloride [155].

A study on transcutaneous immunization (TCI) of anti Aβ1-42 was performed by Matsuo *et al.* The antigen was incorporated within the MN patch and delivered to the skin to induce a Th2-immune response. MN patch delivered the antigen directly to dermal dendritic cells where langerhans cells are present. These cells are antigen-presenting and produce a Th2-dominant immune response highly required to manage AD by Aβ vaccine therapy. TCI minimized the side effect of Th1 cell dominant meningeal encephalitis initiated during the phase II human clinical trials, which signifies it as a better technique for inducing an immune response against AD *via* the transdermal route [156].

Donepezil hydrochloride (DH) is an anticholinesterase inhibitor used to manage moderate to severe forms of dementia. DH has a low passive diffusion characteristic with a log *P* value of 3.08 to 4.11, which was improved by incorporating it into MN. MN can deliver small hydrophilic molecules within the skin barrier, increasing the transdermal delivery of these small hydrophilic molecules. A successful study on DH-mediated MN patch was conducted by Kearney *et al.* to enhance the delivery of donepezil *via* the transdermal route [157]. Similarly, Kim *et al.* also conducted a study to increase the transdermal delivery of DH. DH was encapsulated within the tips of dissolving MN, whereas 78% of the drug was encapsulated within the tips of dissolving MN. The MN tips provided good mechanical stability and delivered the drug four times more than the oral dose. This approach of DH delivery *via* the transdermal route minimizes the side effects of oral dosage forms [158].

A study on MN incorporated with amantadine hydrochloride and pramipexole dihydrochloride was performed by Hoang *et al.* for continuous stimulation of dopamine receptors by minimizing the peak-trough variation through steady-state zero-order drug input. The dopamine agonist pramipexole and amantadine were widely used in the management of PD. To improve the drug efficacy with minimal side effects, an MN patch was formulated and evaluated for an *in-vitro* release study across porcine skin [159].

In recent years, MN drug delivery systems have become an exciting and demanding area of research. However, to transfer the MN concept from the lab bench to a suitable product in the market, various challenges of microneedles must be studied. For microneedles fabrication, expensive instruments are required. Therefore, entering the field of microneedles research and adopting fabrication technology for bulk production is challenging for related industries. Nowadays, licensing for every microneedle product is carried out instead of specific applications, which delays the licensing of microneedle products and slows down microneedle commercialization. To overcome this drawback, regulation for microneedle product licensing must be established. Also, a quality-by-design approach involving the microneedles licensing method should be established by combining cGMP and quality control to encourage commercializing microneedles-based products as pharmaceuticals [160].

## CONCLUSION AND FUTURE PERSPECTIVES

CNS consists of billions of neurons with varied functions and pathological features that maintain normal brain homeostasis. Alteration in these pathological events induces several cascades of events associated with the ND. In that, mitochondrial dysfunction plays a typical role in the progression of ND. Mitochondria are implicated in various cellular activities, including Ca^2+^ balance, ATP production, ROS production, maintenance of neuronal activity, and plasticity. Significant changes in these events cause the induction of sequential pathological events, leading to apoptosis. In recent years, various phytobioactive compounds have shown efficacy in treating oxidative stress induced by mitochondrial dysfunction through *in-vitro* and *in-vivo* studies. These phytobioactive compounds modulate mitochondrial functions such as biogenesis, bioenergetics, calcium homeostasis, membrane potential, and fusion/fission dynamics. Hence, targeting phytobioactive compounds in mitochondria will be an effective treatment strategy for the management of ND, which is one of the researchers' significant tasks. Phytobioactive compounds have poor bioavailability with extensive first-pass effects through oral administration. This minimizes its efficacy in treating the disease condition. To overcome this, MN array technology can be used as a promising drug delivery strategy with the motive of loading phytobioactive compounds in the micron-size needles of MN patches, which will be pierced into the *stratum corneum* layer on the administration of the skin. It avoids the impermeable drawback of TDDS and improves the systemic absorption of phytobioactive compounds. With this approach, phytobioactive compounds can be targeted to the mitochondria of neuronal cells to manage ND effectively. Along with the advantages of decreased pain, discomfort, tissue damage and infection risk, MN technology can provide affordable, accurate and dependable doses with delayed action, which can make them a more acceptable and favorable drug delivery system in the future. The approach may be used in future studies where targeting mitochondria through an MN patch will be a novel drug delivery technique in treating the existing pathology of ND.

## Figures and Tables

**Fig. (1):**
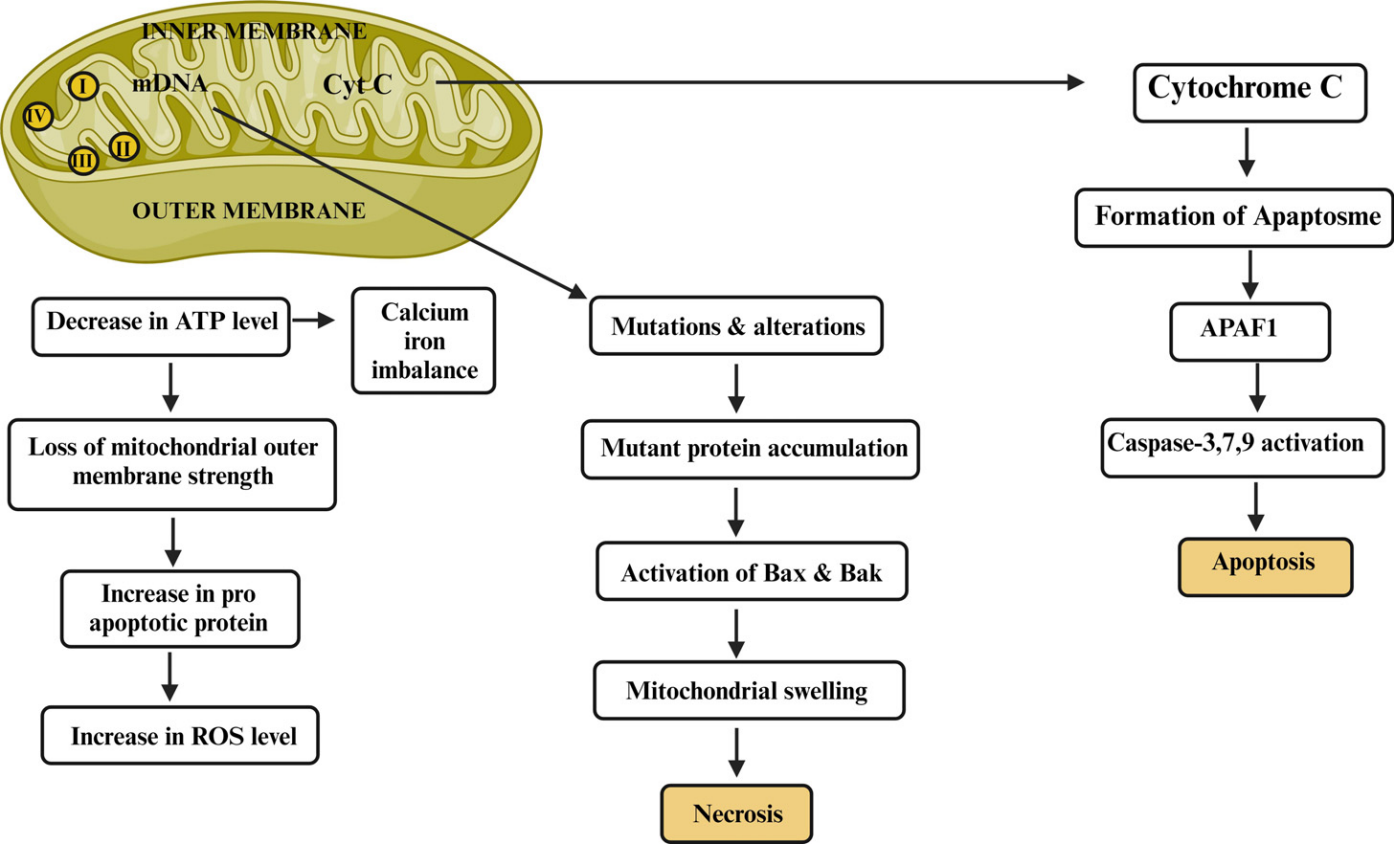
Mechanism involved in mitochondrial dysfunction.

**Fig. (2):**
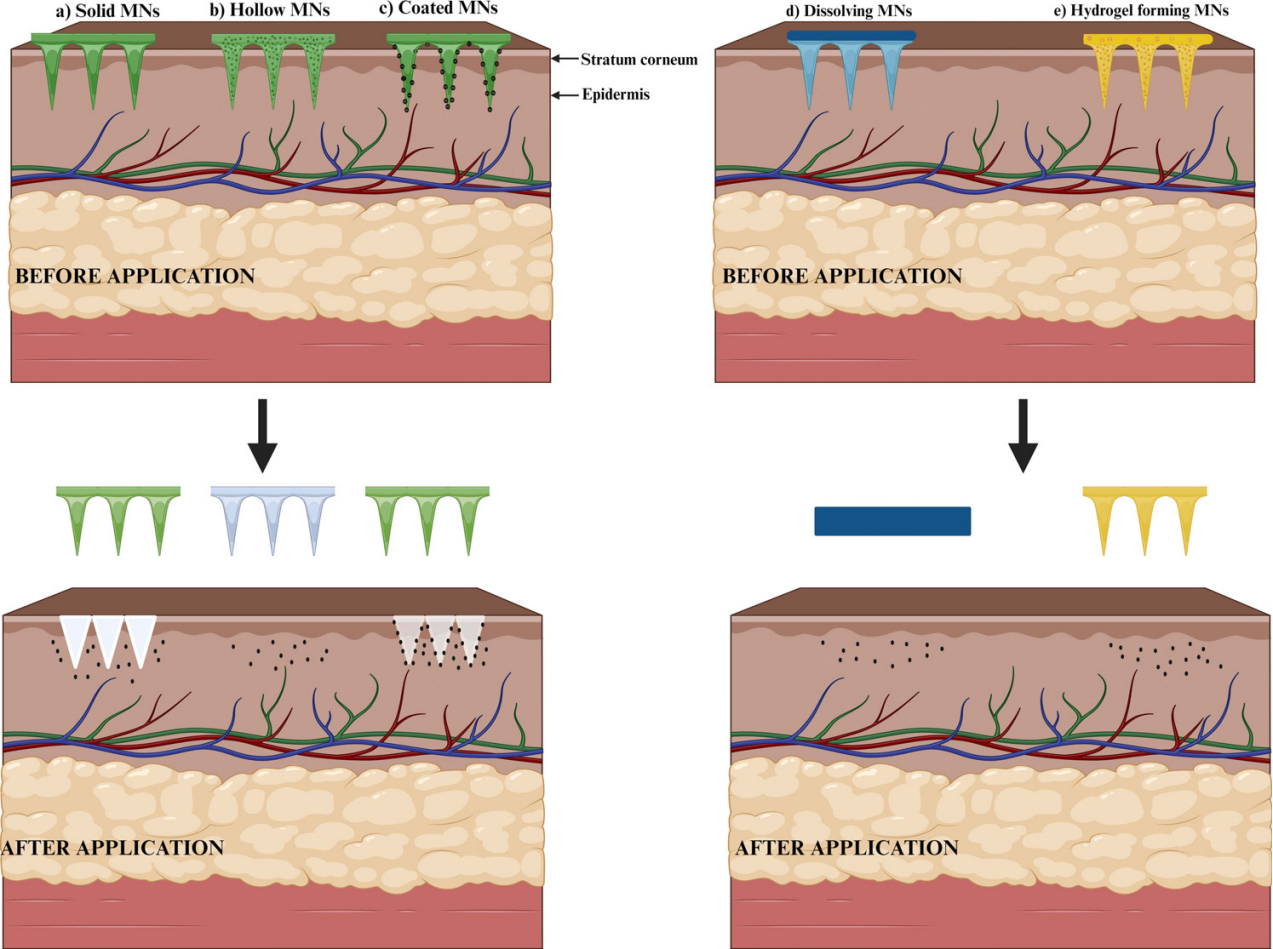
Different types of microneedles (**a**) Solid MN. (**b**) Hollow MN. (**c**) Coated MN. (**d**) Dissolvable MN. (**e**) Hydrogel-forming MN.

**Table 1:** Pathogenesis, symptoms, therapeutic targets, and the relationship with mitochondrial dysfunction in terms of different NDs.

**Sr. No.**	**Type of Disease**	**Pathogenesis**	**Symptoms**	**Therapeutic Targets**	**References**
1.	Alzheimer’s disease	Extracellular and intracellular accumulation of senile plaques and neurofibrillary tangles (NFTs).	Memory loss, progressive deterioration in language function, lack of sensitivity, a steady decrease in everyday living skills, and unusual personality and behavioral changes.	Oxidative stress-induced respiratory chain dysfunction, loss of mitochondrial biogenesis, defects of mitochondrial dynamics, and mtDNA mutations.	[16, 18, 19]
2.	Parkinson’s disease	Accumulation of Lewy bodies, intracellular protein, and Lewy Neuritis.	Stiffness of limbs, slow movement, tremors in hands and other joints, and difficulty in balance and coordination of different body parts and activities.	Targeting Sirtuins (SIRT1, 2, and 3), Nuclear factor erythroid 2-related factor 2 (Nrf2)-antioxidant response element (ARE) pathway, and the mitochondria-endoplasmic reticulum contact sites (MERCs).	[21-23]
3.	Amyotrophic lateral sclerosis	Defects in protein production regulation, hyperactivation of microglia, decreased energy supply from reduced MCT (monocarboxylate transporter) 1 transporter, excitotoxicity, cytoskeletal deficiencies, and RNA synthesis disorders.	Difficulty in swallowing and speaking, body weight loss, muscle weakness, cognitive dysfunction, emotional lability, and fasciculation.	Targeting apoptosis, oxidative stress, electron transport chain, mitophagy/degradation, excitotoxicity, calcium buffering, and anti-inflammation.	[22, 25, 26]
4.	Multiple sclerosis	Inflammation of white and grey matter tissues in CNS.	Weariness, twitches, cognitive difficulties, mood or emotional disabilities, dizziness, convulsion, vision issues.	Targeting metabolism of innate immune cells.	[28, 29, 31]
5.	Huntington’s disease	Expansion of CAG trinucleotide gene expression of the huntingtin gene.	Depression, irritability, obsessive compulsive symptoms, apathy, psychosis, and progressive cognitive decline.	Targeting peroxisome proliferator-activated receptor γ coactivator 1α (PGC-1α), regulation of intracellular calcium homeostasis, antioxidant activity, and reactive oxygen species (ROS) generation.	[32, 35]
6.	Multiple system atrophy	Accumulation of aggregated α-synuclein in oligodendrocytes forming glial cytoplasmic inclusions (GCIs).	Cognitive impairment, mood disorders, and pain.	Targeting oxidative stress and inflammation.	[36-38]

**Table 2:** NDs category, microneedle type, materials, fabrication methods, and drugs used for the treatment of ND.

**Sr. No.**	**Type of ND**	**Microneedle Type**	**Material**	**Method of Fabrication**	**Drug for Use**	**References**
1.	Alzheimer’s disease	Dissolving MN	Sodium hyaluronate	Micromolding technology	Huperzine A	[151]
2.	Parkinson’s disease	Dissolving MN	Polyvinyl alcohol	Micromolding technology-poly-di-methyl-siloxane (PDMS) mould	Curcumin	[152]
3.	Parkinson’s disease	Dissolving MN	Polyvinyl alcohol	Micromolding technology-poly-di-methyl-siloxane (PDMS) mould	Bacopa moneri	[153]
4.	Parkinson’s disease	Coated MN	Gelatin-methacryloy	Micromolding technology	Levodopa	[154]
5.	Parkinson’s disease	Hollow MN	AdminPatch array 600-mm microneedles	Readymade	Ropinirole hydrochloride	[155]
6.	Alzheimer’s disease	Dissolving MN	Sodium hyaluronate	Micromolding technology	Amyloid β vaccine	[156]
7.	Alzheimer’s disease	Hydrogel-forming MN	Gantrez S-97, Poly ethylene glycol, Sodium carbonate	Micromolding technology	Donepezil hydrochloride	[157]
8.	Alzheimer’s disease	Dissolving MN	Hydroxy-propyl-methyl-cellulose (HPMC)	Micromolding technology-poly-di-methyl-siloxane (PDMS) mold	Donepezil hydrochloride	[158]
9.	Parkinson’s disease	Solid MN	Stainless steel microneedle rollers	Readymade	Amantadine hydrochloride and Pramipexole dihydrochloride	[159]

## LIST OF ABBREVIATIONS

5-ALA: 5-Aminolevulinic Acid
AD: Alzheimer’s Disease
ADHD: Attention Deficit Hyperactivity Disorder
ALS: Amyotrophic Lateral Sclerosis
BPD: Bipolar Disorder
DMN: Default Mode Network
ETC: Electron Transport Chain
GCIs: Glial Cytoplasmic Inclusions
HD: Huntington’s Disease
LDLs: Low-density Lipoproteins
MCT: Monocarboxylate Transporter
MDD: Major Depressive Disorder
MEMS: Microelectromechanical Devices
MN: Microneedle
MS: Multiple Sclerosis
MSA: Multiple System Atrophy
ND: Neurodegenerative Disease
NFTs: Neurofibrillary Tangles
PD: Parkinson’s Disease
ROS: Reactive Oxygen Species
SCZ: Schizophrenia
TDP43: TAR DNA-binding Protein 43
